# Supplementary material for: The Genetics of Polycystic Ovary Syndrome: An Overview of Candidate Gene Systematic Reviews and Genome-Wide Association Studies
Source: J Clin Med. 2019 Oct 3;8(10):1606. doi: 10.3390/jcm8101606 (PMC6832583; doi:10.3390/jcm8101606)
Supplement: Supplementary file 1 [file jcm-08-01606-s001.pdf]

Supplementary Data

Supplemental table 1- Participant, Intervention, Comparison, outcomes and studies (PICOs) framework

	**Participants**	**Interventions**	**Comparisons**	**Outcomes**	**Study design**	**Limits**
**Inclusion Criteria**	-Any population where PCOS is PRIMARY FOCUS.<br><br>-Can be interventions in PCOS, descriptions/characterisations of PCOS or comparisons of women with and without PCOS	Any systematic review with or without a meta-analysis<br><br>-Review must include a search strategy containing at least key words or terms<br>-Review must include the number of identified and included articles<br>-Review must include some form of article quality appraisal	N/A	ANY systematic review with or without meta-analysis.<br><br>-Following article screening and data extraction the reviews will be grouped by theme: genetics, assessment, fertility treatment, Non-fertility treatment, lifestyle management, complementary or alternative medicines/therapies.	Any systematic review with or without a meta-analysis	-English Only<br>-Humans<br>-Published 2009 to current
**Exclusion criteria**	PCOS is a secondary outcome (Not main focus of the SR)		N/A		RCTs<br>Cohort studies<br>Case-control<br>Cross sectional<br>Grey literature<br>Longitudinal<br>Qualitative<br>Case study<br>Editorial<br>Narrative review	-Not written in English<br>-Animal Models<br>-Published prior to 2009

Supplemental Table 2: Search strategy in MEDLINE

1. exp Polycystic Ovary Syndrome/
2. Polycystic Ovar$.tw
3. pco.tw or pcos.tw
4. (sclerocystic adj3 ovar$).tw
5. stein leventhal.tw
6. or/1–5
7. Meta-Analysis as Topic/
8. meta analy$.tw
9. metaanaly$.tw
10. Meta-Analysis/
11. (systematic adj (review$1 or overview$1)).tw.
12. exp Review Literature as Topic/
13. or/7-12
14. cochrane.ab.
15. embase.ab.
16. (psychlit or psyclit).ab.
17. (psychinfo or psycinfo).ab.
18. (cinahl or cinhal).ab.
19. science citation index.ab.
20. bids.ab.
21. cancerlit.ab.
22. or/14-21
23. reference list$.ab.
24. bibliograph$.ab.
25. hand-search$.ab.
26. relevant journals.ab.
27. manual search$.ab.
28. or/23-27
29. selection criteria.ab.
30. data extraction.ab.
31. 29 or 30
32. Review/
33. 31 and 32
34. Comment/
35. Letter/
36. Editorial/
37. animal/
38. human/
39. 37 Not (37 and 38)
40. or/34-36,39
41. 13 or 22 or 28 or 33
42. 6 and 41
43. 42 Not 40

Supplementary table 3: Articles excluded on full text screening.

Author	Title	Notes
Abu Hashim 2016	Twenty years of ovulation induction with metformin for PCOS; what is the best available evidence?	Didn't include quality assessment
Al Khalifah 2015	The effectiveness and safety of treatments used for polycystic ovarian syndrome management in adolescents: a systematic review and network meta-analysis protocol	Protocol only
Amihaesei, 2016	Polycystic Ovary Syndrome, A Complex Entity Involving Reproductive and Metabolic Impairments: A Systematic Review.	Didn't include quality assessment, number of articles extracted on search, or search strategy
Atiomo 2009	Proteomic biomarkers for the diagNosis and risk stratification of polycystic ovary syndrome: a systematic review	Didn't include quality assessment
Bacopoulou 2017	Vitamin D predictors in polycystic ovary syndrome: a meta-analysis.	Didn't include quality assessment
Bagos 2009	PlasmiNogen activator inhibitor-1 4G/5G and 5,10-methylene-tetrahydrofolate reductase C677T polymorphisms in polycystic ovary syndrome	Didn't include quality assessment or number of articles extracted on search
Bahri 2019	PREGNANCY COMPLICATIONS ACROSS PHENOTYPES IN POLYCYSTIC OVARY SYNDROME: A META-ANALYSIS	Abstract only
Bao 2016	Association of DENND1A Gene Polymorphisms with Polycystic Ovary Syndrome: A Meta-Analysis	Didn't include quality assessment
BaraNova 2011	Systematic review: association of polycystic ovary syndrome with metabolic syndrome and Non-alcoholic fatty liver disease	Didn't include number of articles extracted on search or quality assessment
Barba 2009	The effects of metformin on endogeNous androgens and SHBG in women: a systematic review and meta-analysis	Not primarily focused on PCOS
Bayram 2010	Pulsatile gonadotrophin releasing hormone for ovulation induction in subfertility associated with polycystic ovary syndrome	Although Cochrane assessed content as up to date in 2010, search was conducted in 2003 so deemed this as meeting prior to 2009 exclusion criteria
Birch Petersen 2016	MoNo-ovulation in women with polycystic ovary syndrome: a clinical review on ovulation induction	Didn't include search terms or number of articles extracted on search
Bouza-Alvarez	Safety and efficacy of metformin in improving clinical, hormonal and metabolic features of polycystic ovary syndrome. Systematic review and meta-analysis	? HTA, bulk of article in Spanish
Bronstein 2011	Age of onset of polycystic ovarian syndrome in girls may be earlier than previously thought	Didn't include quality assessment
Cahill 2009	PCOS	Didn't include search terms or number of articles extracted on search
Cahill 2015	Polycystic ovary syndrome (PCOS): metformin	Didn't include search terms or number of articles extracted on search
Cai 2014	Association between fat mass- and obesity-associated (FTO) gene polymorphism and polycystic ovary syndrome: a meta-analysis	Didn't include quality assessment
Carlus 2016	Is MTHFR 677 C>T Polymorphism Clinically Important in Polycystic Ovarian Syndrome (PCOS)? A Case-Control Study, Meta-Analysis and Trial Sequential Analysis	Didn't include quality assessment
Chae 2017	Metabolic features of adult and adolescent first-degree relatives of women with polycystic ovary syndrome: a systematic review and meta-analysis	Conference abstract
Chen 2014	Two follicle-stimulating hormone receptor polymorphisms and polycystic ovary syndrome risk: a meta-analysis	Didn't include quality assessment
Chittenden 2009	Polycystic ovary syndrome and the risk of gynaecological cancer: a systematic review	Didn't include quality assessment
Conte 2015	Mental Health and Physical Activity in Women with Polycystic Ovary Syndrome: A Brief Review	Didn't include quality assessment

Costello 2010	Insulin-sensitising drugs versus the combined oral contraceptive pill for hirsutism, acne and risk of diabetes, cardiovascular disease, and endometrial cancer in polycystic ovary syndrome	No update of search strategy since 2006
Day 2018	Large-scale geNome-wide meta-analysis of polycystic ovary syndrome suggests shared genetic architecture for different diagNosis criteria	Not a systematic review
de Medeiros 2017	Risks, benefits size and clinical implications of combined oral contraceptive use in women with polycystic ovary syndrome	Didn't include quality assessment, number of articles extracted on search
Delitala 2017	Polycystic ovary syndrome, adipose tissue and metabolic syndrome	Narrative review
Dewailly 2016	Interactions between androgens, FSH, anti-Müllerian hormone and estradiol during folliculogenesis in the human Normal and polycystic ovary	Didn't include quality assessment, number of articles extracted on search
Dissemination	Overweight in polycystic ovary syndrome. An update on evidence based advice on diet, exercise and metformin use for weight loss	Didn't include number of articles extracted on search or quality assessment
Dissemination	N-acetylcysteine for treating women with clomiphene citrate resistant polycystic ovary syndrome: a systematic review	Not in English
Dissemination	Rosiglitazone versus metformin for polycystic ovary syndrome: a systematic review (Provisional abstract)	Not in English
Dissemination 2012	Efficacy of iNositol in women with polycystic ovary syndrome and desire for children: systematic review and meta-analysis	Not in English
Dissemination 2015	Letrozole for ovulation induction in women with polycystic ovarian syndrome: a systematic analysis	Not in English
Dissemination 2015	Thiazolidinediones combined with metformin in treatment of polycystic ovary syndrome: a systematic review	Not in English
Dissemination 2016	Laparoscopic surgery versus laparotomy for women with polycystic ovarian syndrome: a systematic review.	Not in English
Dokas 2011	Increased risk for abNormal depression scores in women with polycystic ovary syndrome: a systematic review and meta-analysis	Didn't include quality assessment
Du 2010	Two FSHR variants, haplotypes and meta-analysis in Chinese women with premature ovarian failure and polycystic ovary syndrome	Didn't include search terms, quality assessment or number of articles extracted on search
Du 2013	The relationship between thyroiditis and polycystic ovary syndrome: a meta-analysis	Didn't include quality assessment
Eckmann 2009	Aromatase inhibitors for ovulation and pregnancy in polycystic ovary syndrome	Didn't include number of articles extracted on search or quality assessment
Escobar-Morreale 2011	Circulating inflammatory markers in polycystic ovary syndrome: a systematic review and metaanalysis	Didn't include quality assessment
Escobar-Morreale 2016	Health-related quality of life in polycystic ovary syndrome patients: A systematic review	did Not address PCOS co-morbidities
Eyvazzadeh 2009	The role of the endogeNous opioid system in polycystic ovary syndrome	Not a systematic review
Fan 2013	Association between the (TAAAA)n SHBG polymorphism and PCOS: a systematic review and meta-analysis	Didn't include quality assessment
Farquhar 2009	Laparoscopic ovarian diathermy versus metformin for women with polycystic ovarian syndrome	
Fazelian 2017	Chromium supplementation and polycystic ovary syndrome: A systematic review and meta-analysis	Didn't include a quality assessment
Fazleen 2018	Risk of metabolic syndrome in adolescents with polycystic ovarian syndrome: A systematic review and meta-analysis	Did Not include search strategy
Fernandez 2011	Ovarian drilling for surgical treatment of polycystic ovarian syndrome: a comprehensive review	Didn't include quality assessment
Filippou 2017	Is foetal hyperexposure to androgens a cause of PCOS?	Narrative review

Frary 2016	The effect of dietary carbohydrates in women with polycystic ovary syndrome: a systematic review.	Didn't include quality assessment
Fu 2014	Association of methylenetetrahydrofolate reductase gene C677T polymorphism with polycystic ovary syndrome risk: a systematic review and meta-analysis update	Didn't include quality assessment
Galazis 2012	Proteomic biomarkers for ovarian cancer risk in women with polycystic ovary syndrome: a systematic review and biomarker database integration	Exclude, PCOS SR component update of Atiomo 2008/9 with No quality assessment
Galazis 2012	Metabolomic biomarkers of impaired glucose tolerance and type 2 diabetes mellitus with a potential for risk stratification in women with polycystic ovary syndrome	Not primarily focused on PCOS
Galazis 2013	Proteomic biomarkers of endometrial cancer risk in women with polycystic ovary syndrome: a systematic review and biomarker database integration	Exclude, PCOS SR component update of Atiomo 2008/9 with No quality assessment
Galazis 2013	Proteomic biomarkers of preterm birth risk in women with polycystic ovary syndrome (PCOS): a systematic review and biomarker database integration	Exclude, PCOS SR component update of Atiomo 2008/9 with No quality assessment
Gao 2012	Association of the T45G and G276T polymorphisms in the adiponectin gene with PCOS: A meta-analysis	Didn't include quality assessment or number of articles extracted on search
Garg 2016	Relationship between Advanced Glycation End Products and Steroidogenesis in PCOS	Didn't include quality assessment, number of articles extracted on search
Groth 2010	Adiponectin and Polycystic Ovary Syndrome	Didn't include number of articles extracted on search or quality assessment
Gu 2016	The association between paraoxonase 1 gene polymorphisms and polycystic ovarian syndrome	Didn't include a quality assessment
Gui 2017	ardiovascular autoNomic dysfunction in women with polycystic ovary syndrome: a systematic review and meta-analys	Didn't include a quality assessment
Haoula 2012	Evaluating the association between endometrial cancer and polycystic ovary syndrome	Didn't include quality assessment
Harris 2018	Polycystic Ovary Syndrome, OligomeNorrhea, and Risk of Ovarian Cancer Histotypes: Evidence from the Ovarian Cancer Association Consortium.	Narrative review
He 2012	A meta-analysis on the association between PPAR-gamma Pro12Ala polymorphism and polycystic ovary syndrome	Didn't include quality assessment
Huang 2012	Four polymorphisms of the CAPN 10 gene and their relationship to polycystic ovary syndrome susceptibility: a meta-analysis	Didn't include quality assessment or number of articles extracted on search
Ioannidis 2010	Polymorphisms of the insulin receptor and the insulin receptor substrates genes in polycystic ovary syndrome: a Mendelian randomization meta-analysis	Didn't include quality assessment or number of articles extracted on search
Jalilian 2015	Prevalence of polycystic ovary syndrome and its associated complications in Iranian women: A meta-analysis	Didn't include quality assessment
Janci 2012	Polycystic Ovarian Syndrome: Metformin or Thiazolidinediones for Cardiovascular Risk Reduction?	Didn't include number of articles extracted on search or quality assessment
Jia 2013	Association of angiotensin-converting enzyme gene insertion/deletion polymorphism with polycystic ovary syndrome: a meta-analysis	Didn't include quality assessment
Jia 2014	Association between retiNol-binding protein 4 and polycystic ovary syndrome: a meta-analysis	Didn't include quality assessment
Johnson 2011	Metformin is a reasonable first-line treatment option for Non-obese women with infertility related to aNovulatory polycystic ovary syndrome--a meta-analysis of randomised trials	Didn't include search terms, number of articles extracted on search or quality assessment
Kelly 2011	Insulin-like growth factor binding protein-1 in PCOS: a systematic review and meta-analysis	Didn't include quality assessment
Khan 2015	Overlap of proteomics biomarkers between women with pre-eclampsia and PCOS: a systematic review and biomarker database integration	Exclude, PCOS SR component update of Atiomo 2008/9 with No quality assessment

Kjerulff 2011	Pregnancy outcomes in women with polycystic ovary syndrome: a metaanalysis	Didn't include quality assessment
Kong 2015	Impact of Treatment with Metformin on Adipocytokines in Patients with Polycystic Ovary Syndrome: A Meta-Analysis	Didn't include quality assessment
Krul-Poel 2013	The role of vitamin D in metabolic disturbances in polycystic ovary syndrome: a systematic review	Didn't include quality assessment
Lai 2014	Chinese Herbal Medicine for OligomeNorrhoea and AmeNorrhoea in Polycystic Ovary Syndrome: A Systematic Review and Meta-Analysis	Abstract only
Lakkakula 2013	Genetic variants associated with insulin signaling and glucose homeostasis in the pathogenesis of insulin resistance in polycystic ovary syndrome: a systematic review	Didn't include search terms, quality assessment or number of articles extracted on search
Lautatzis 2013	Efficacy and safety of metformin during pregnancy in women with gestational diabetes mellitus or polycystic ovary syndrome: a systematic review	Didn't include search terms
Lee 2014	PlasmiNogen activator inhibitor-1 4G/5G and the MTHFR 677C/T polymorphisms and susceptibility to polycystic ovary syndrome: a meta-analysis	Didn't include quality assessment or number of articles extracted on search
Lim 2010	Current evidence of acupuncture on polycystic ovarian syndrome.	Didn't include search terms, number of articles extracted on search or quality assessment
Lin 2013	Androgen receptor gene polymorphism and polycystic ovary syndrome	Didn't include quality assessment
Lin 2014	Is a GnRH antagonist protocol better in PCOS patients? A meta-analysis of RCTs	Didn't include quality assessment
Liu 2014	PlasmiNogen activator inhibitor-1 -675 4G/5G polymorphism and polycystic ovary syndrome risk: a meta analysis	Didn't include quality assessment
Liu 2016	Meta-analysis of the correlation between the TNF-alpha308G/A polymorphism and polycystic ovary syndrome	Didn't include quality assessment
Liu 2017	Association between fat mass and obesity associated (FTO) gene rs9939609 A/T polymorphism and polycystic ovary syndrome: a systematic review and meta-analysis	Didn't include a quality assessment
Louwers 2013	Cross-ethnic meta-analysis of genetic variants for polycystic ovary syndrome	Didn't include quality assessment or number of articles extracted on search
Mancini 2011	Gonadotrophin-releasing hormone-antagonists vs long agonist in in-vitro fertilization patients with polycystic ovary syndrome: a meta-analysis	Didn't include quality assessment
Misso 2012	Status of clomiphene citrate and metformin for infertility in PCOS	Not a systematic review
Misso 2015	Metformin in women with PCOS, cons	Not a systematic review
Morris 2016	What does a diagNostic label of 'polycystic ovary syndrome' really mean in adolescence? A review of current practice recommendations	Didn't include quality assessment
Naderpoor 2015	Metformin and lifestyle modification in polycystic ovary syndrome: systematic review and meta-analysis	Didn't include search terms
Nahuis 2011	Metformin co-administration during follicle stimulating hormone ovulation induction with timed intercourse or intra-uterine insemination for subfertility associated with polycystic ovary syndrome	
Nahuis 2013	The basic fertility workup in women with polycystic ovary syndrome: a systematic review	Didn't include quality assessment
Niafar 2016	A systematic review of GLP-1 agonists on the metabolic syndrome in women with polycystic ovaries	Didn't include quality assessment
Nicholson 2010	Effectiveness of long-term (twelve months) Nonsurgical weight loss interventions for obese women with polycystic ovary syndrome: a systematic review	Didn't include quality assessment
Omabe 2018	Emerging Metabolomics Biomarkers of Polycystic Ovarian Syndrome; Targeting the Master Metabolic Disrupters for DiagNosis and Treatment	Narrative review
Palomba 2015	Pregnancy complications in women with polycystic ovary syndrome	Didn't include number of articles extracted on search or quality assessment

Parsanezhad 2009	Surgical ovulation induction in women with polycystic ovary syndrome: a sytematic review	Didn't include number of articles extracted on search or quality assessment
Peitsidis 2010	Role of vascular endothelial growth factor in women with PCO and PCOS: a systematic review	Didn't include quality assessment
Peng 2014	The association between androgen receptor gene CAG polymorphism and polycystic ovary syndrome: a case-control study and meta-analysis	Didn't include quality assessment
Qin 2013	Obstetric complications in women with polycystic ovary syndrome: a systematic review and meta-analysis	Didn't include quality assessment
Rajender 2013	Androgen receptor CAG repeats length polymorphism and the risk of polycystic ovarian syndrome (PCOS)	Didn't include quality assessment
Reis 2017	Vitamin D receptor polymorphisms and the polycystic ovary syndrome: A systematic review	Narrative review
Ren 2014	[A meta-analysis on acupuncture treatment of polycystic ovary syndrome]	Not in English
Rocca 2015	Polycystic ovary syndrome: chemical pharmacotherapy	Not a systematic review
Rocha 2017	Non-alcoholic fatty liver disease in women with polycystic ovary syndrome: systematic review and meta-analysis.	Didn't include a quality assessment
Saha 2013	N-acetyl cysteine in clomiphene citrate resistant polycystic ovary syndrome: A review of reported outcomes	Didn't include quality assessment
San-Millan 2010	The role of genetic variation in peroxisome proliferator-activated receptors in the polycystic ovary syndrome (PCOS): an original case-control study followed by systematic review and meta-analysis of existing evidence	Didn't include quality assessment
Showell 2016	INositol for subfertile women with polycystic ovary syndrome	
Sirmans 2012	Polycystic ovary syndrome and chronic inflammation: pharmacotherapeutic implications	Didn't include number of articles extracted on search or quality assessment
Skowronska 2016	The role of vitamin D in reproductive dysfunction in women - a systematic review.	Didn't include quality assessment, number of articles extracted on search
Song 2014	Lack of association of INS VNTR polymorphism with polycystic ovary syndrome: a meta-analysis	Didn't include quality assessment
Sun 2013	Effect of metformin on ovulation and reproductive outcomes in women with polycystic ovary syndrome: a meta-analysis of randomized controlled trials	Didn't include quality assessment
Taghavi 2015	Type 1 Diabetes and Polycystic Ovary Syndrome: Systematic Review and Meta-analysis	Not primarily focused on PCOS
Tang 2009	WITHDRAWN: Insulin-sensitising drugs for polycystic ovary syndrome	Withdrawn from publication as error in citation
Tang 2010	Ultrasound-guided transvaginal ovarian needle drilling for clomiphene-resistant polycystic ovarian syndrome in subfertile women	
Tang 2012	Association of Pro12Ala polymorphism in peroxisome proliferator-activated receptor gamma with polycystic ovary syndrome: a meta-analysis	Didn't include quality assessment
Tang 2015	Insulin receptor substrate-1 (IRS-1) rs1801278G>A polymorphism is associated with polycystic ovary syndrome susceptibility: a meta-analysis	Didn't include quality assessment
Tang 2017	Circulating omentin-1 levels in women with polycystic ovary syndrome: a meta-analysis	Didn't include a quality assessment
Thethi 2015	Role of Insulin Sensitizers on Cardiovascular Risk Factors in Polycystic Ovarian Syndrome: A Meta-Analysis	"Reviewers worked independently and in duplicate to determine the methodological quality" but No details given
Tomlinson 2010	Type 2 diabetes and cardiovascular disease in polycystic ovary syndrome: what are the risks and can they be reduced?	Didn't include search terms or number of articles extracted on search

Toulis 2011	Meta-analysis of cardiovascular disease risk markers in women with polycystic ovary syndrome	Didn't include quality assessment
Tsikouras 2015	Features of Polycystic Ovary Syndrome in adolescence	Didn't include number of articles extracted on search or quality assessment
Unfer 2012	Effects of myo-iNositol in women with PCOS: a systematic review of randomized controlled trials	Didn't include quality assessment
Wang 2012	[Therapeutic effect of metformin for clomiphene-resistant infertility patients with polycystic ovary syndrome: a systematic analysis]	Not in English
Wang 2012	Negative association between androgen receptor gene CAG repeat polymorphism and polycystic ovary syndrome? A systematic review and meta-analysis	Didn't include quality assessment
Wang 2015	4G/5G polymorphism of plasmiNogen activator inhibitor-1 gene is associated with polycystic ovary syndrome in Chinese patients: a meta-analysis	Didn't include quality assessment or number of articles extracted on search
Wang 2017	Pregnancy complications among women with polycystic ovary syndrome in China: a Meta-analysis	Not in English
Wang 2017	The role of AMH and its receptor SNP in the pathogenesis of PCOS	Didn't include a quality assessment
Wang 2018	Higher circulating irisin levels in patients with polycystic ovary syndrome:a meta-analysis.	Didn't include a quality assessment
Wild 2010	Assessment of cardiovascular risk and prevention of cardiovascular disease in women with the polycystic ovary syndrome: a consensus statement by the Androgen Excess and Polycystic Ovary Syndrome (AE-PCOS) Society	Not a systematic review
Wild 2011	Lipid levels in polycystic ovary syndrome: systematic review and meta-analysis	Didn't include quality assessment
Wojciechowski 2012	Impact of FTO geNotypes on BMI and weight in polycystic ovary syndrome: a systematic review and meta-analysis	Didn't include quality assessment
Wu 2016	Acupuncture for treating polycystic ovary syndrome: guidance for future randomized controlled trials	Didn't include number of articles extracted on search
Xian 2012	ADIPOQ gene polymorphisms and susceptibility to polycystic ovary syndrome: a HuGE survey and meta-analysis	Didn't include quality assessment
Xiao 2011	Effectiveness of GnRH antagonist in vitro fertilization-embryo transfer (IVF-ET) in PCOS patients: a systematic review	Not in English
Xie 2013	Microsatellite polymorphism in the fibrillin 3 gene and susceptibility to PCOS: a case-control study and meta-analysis	Didn't include quality assessment or number of articles extracted on search
Xu 2014	Effect of metformin on serum interleukin-6 levels in polycystic ovary syndrome: a systematic review	Didn't include quality assessment
Yu 2014	Polymorphisms of pentanucleotide repeats (tttta)n in the promoter of CYP11A1 and their relationships to polycystic ovary syndrome (PCOS) risk: a meta-analysis	Didn't include quality assessment
Yu 2017	Comparative effectiveness of 9 ovulation-induction therapies in patients with clomiphene citrate-resistant polycystic ovary syndrome: a network meta-analysis.	Did Not include search strategy
Zhang 2012	Association between the Pro12Ala polymorphism of PPAR-gamma gene and the polycystic ovary syndrome: a meta-analysis of case-control studies	Didn't include quality assessment
Zhang 2014	The -675 4G/5G polymorphism in the PAI-1 gene may Not contribute to the risk of PCOS	Didn't include quality assessment or number of articles extracted on search
Zhang 2015	Peroxisome proliferator-activated receptor gamma rs1801282 C>G polymorphism is associated with polycystic ovary syndrome susceptibility: a meta-analysis involving 7,069 subjects	Didn't include quality assessment
Zhang 2018	Effect of microecological preparation supplementation on woman with polycystic ovary syndrome: A meta-analysis protocol	Protocol only

Supplementary table 4: Characteristics of included systematic reviews assessing the genetics of PCOS.

Author	Publication date	Languages included in SR	Sample size of SR (n= participants)	Types of primary studies included	SR methodology	QA methodology and authors judgement of quality (if available) of primary studies
Chen et al. [1]	2018	No restriction	PCOS 512;<br>Controls 606	Case-control or cohort	Yes- PRISMA	NOS-6.33/9<br>(Acceptable)
Feng et al.[2]	2015	No restriction	PCOS 17 460;<br>Controls 23 845	Case-control or cohort	Yes- PRISMA	NOS- 6.75/9<br>(did Not specify if considered high or low quality)
Gao et al. [3]	2016	No restriction	PCOS 15 074<br>Controls 113 997	Case-control	No	NOS- 6.6/9<br>(did Not specify if considered high or low quality)
Guo et al.[4]	2015	No restriction	PCOS 1 665;<br>Controls 1 687	Case-control	Yes- PRISMA	NOS- High quality
Jia et al.[5]	2012	English	T45G SNP<br>PCOS 1 104;<br>Controls 1 717<br><br>G276T<br>PCOS 823;<br>Controls 982	Case-control	No	Modified scoring system - Low quality
Li et al.[6]	2012	No restriction	PCOS 1321;<br>Control 1017	Case-control	No	QA methodology Not specified.<br>Quality of primary studies was satisfactory.
Liao et al. [7]	2018	No restriction	PCOS: 2249<br>Controls: 1977	Case-control	No	NOS (good to fair)
Liu et al. [8]	2018	English or Chinese	rs1501299 G/T<br>PCOS: 2602<br>CON: 3773<br><br>rs2241766 T/G<br>PCOS: 2358<br>CON: 4034	Case-control	Yes- PRISMA	NOS (High quality)
Pabalan et al. [9]	2016	No restrictions	Not described	Case-control	Yes- PRISMA	Clark-Baudouin Score (High quality)
Qiu et al.[10]	2015	English and Chinese	PCOS 2670;<br>Control 7752	Case-control	No	CASP & STREGA- Medium to high quality
Ramos et al.[11]	2015	No restrictions	PCOS 1892;<br>Control 2695	Case-control and cross-sectional	Yes- PRISMA	NOS- Range 5 to 7
Ruan et al.[12]	2012	English	PCOS 1877;<br>Control 2 444	Case-control and cross-sectional	No	QA methodology Not specified a
Shen et al.[13]	2013	No Restriction	PCOS 2123;<br>Control 3612	Case-control	No	NOS- Good quality
Shen et al.[14]	2013	No Restriction	PCOS 521;<br>Control 515	Case-control	No	STROBE- Moderate to high quality
Shen et al.[15]	2014	No Restriction	PCOS 1571;<br>Control 1918	Case-control	No	NOS- Poor to good Quality
Shen et al.[16]	2014	No Restriction	PCOS 2458;<br>Control 5109	Clinical cohort and case-control	No	NOS- Poor to good quality
Shi et al.[17]	2016	English or Chinese	PCOS 2975;<br>Control 3011	Case-control	No	NOS- Low to high Quality
Wang et al.[18]	2015	English or Chinese	PCOS 351;<br>Control 464	Case-control	No	10 point scoring system- Fair quality

Wu et al.[19]	2015	No restriction	TNFα 308G > A:<br>582 PCOS, 563 control<br>TNFα -805C > T:<br>87 PCOS, 115 control;<br>TNFα -1031 T > C:<br>500 PCOS, 450 control;<br>IL-1A -889C > T<br>310 PCOS, 279 controls IL-1B -511C > T<br>482 PCOS, 421 control IL-1B +3953C > T<br>223 PCOS, 188 control<br>IL-6 -174G > C<br>416 PCOS, 569 control<br>IL-10 -819C > T<br>91 PCOS, 75 control<br>IL-10 -1082A > G<br>188 PCOS, 170 control IL-18 -607C > A<br>118 PCOS, 79 control; IL-18 -137G > C<br>244 PCOS, 192 controls	Case-control	Yes- PRISMA	NOS- Low to high quality
Yan et al.[20]	2014	English and Chinese	PCOS 1075;<br>Control 2878	Case-control	Yes- PRISMA	NOS- Low to high Quality
Zhang et al.[21]	2013	No restriction	PCOS 1144; Control 1409	Not stated	Yes- MOOSE	QA methodology Not specified a

CASP, Critical Appraisal Skills Programme; CBS, Clark-Baudouin Score; IL, Interleukin; MOOSE, Meta-analysis Of Observational Studies in Epidemiology; NOS, Newcastle Ottawa Scale; PCOS: Polycystic ovary syndrome; PRISMA, Preferred Reporting Items for Systematic Reviews and Meta-Analyses; QA, Quality Appraisal; SNP, Single Nucleotide Polymorphisms; STREGA, Strengthening The Reporting of Genetic Association studies; STROBE, Strengthening the Reporting of Observational Studies in Epidemiology; TNF-α, Tumor Necrosis Factor-Alpha.

a Li et al. (2012), Ruan et al. (2012), and Zhang et al. (2013) completed quality appraisal and therefore fulfilled the PICO inclusion criteria however did Not specify by which method they assessed quality or indicate the quality of the primary studies.

Supplementary table 5: AMSTAR appraisal of included systematic reviews [22]

Study	PICO	*Apriori* design*	Study selection	Comprehensive literature search *	Study selection in duplicate	Data extraction in duplicate	List and justification of excluded studies*	Characteristics (must include recruitment source)	Assess primary studies ROB *	Funding	Studies combined using appropriate methods *	Assessed ROB	Account for ROB when interpreting results*	ROB addressed	Publication bias tested*	Conflict of interest	AMSTAR2 quality
**Chen et al 2018 [1]**	**Yes**	**Yes**	**Yes**	**Yes**	**UA**	**Yes**	**Partial**	**Partial**	**Yes**	**No**	**Yes**	**Yes**	**Yes**	**Yes**	**Yes**	**Yes**	**Moderate**
**Feng et al 2015 [2]**	**Yes**	**Yes**	**Yes**	**Yes**	**UA**	**Yes**	**Yes**	**Partial**	**Yes**	**No**	**Yes**	**Yes**	**Yes**	**Yes**	**Yes**	**Yes**	**Moderate**

**Gao et al 2016 [3]**	**Yes**	**Partial**	**Yes**	**Partial**	**Yes**	**Yes**	**Partial**	**Partial**	**Yes**	**No**	**Yes**	**Yes**	**Yes**	**Yes**	**Yes**	**Yes**	**High**
**Guo et al, 2015 [4]**	**Yes**	**Partial**	**Yes**	**Yes**	**Yes**	**Yes**	**Partial**	**Partial**	**Yes**	**No**	**Yes**	**Yes**	**Yes**	**Yes**	**Yes**	**Yes**	**High**
**Jia et al 2012 [5]**	**Yes**	**Partial**	**Yes**	**No**	**UA**	**Yes**	**Partial**	**Partial**	**Yes**	**No**	**Yes**	**Yes**	**Yes**	**Yes**	**Yes**	**Yes**	**Low**
**Li et al. 2012 [6]**	**Yes**	**Partial**	**Yes**	**Yes**	**Yes**	**Yes**	**Partial**	**Partial**	**No**	**No**	**Yes**	**Yes**	**Yes**	**Yes**	**Yes**	**No**	**Low**
**Liao et al 2018 [7]**	**Yes**	**Partial**	**Yes**	**Yes**	**Yes**	**Yes**	**Partial**	**Yes**	**Yes**	**No**	**Yes**	**Yes**	**Yes**	**Yes**	**Yes**	**Yes**	**Moderate**
**Liu et al. 2018 [8]**	**Yes**	**Partial**	**Yes**	**Yes**	**UA**	**Yes**	**Partial**	**Partial**	**Yes**	**No**	**Yes**	**Yes**	**Yes**	**Yes**	**Yes**	**Yes**	**Moderate**
**Pabalan et al. 2016 [9]**	**Yes**	**Partial**	**Yes**	**Yes**	**UA**	**Yes**	**Partial**	**Partial**	**Yes**	**No**	**Yes**	**Yes**	**Yes**	**Yes**	**No**	**Yes**	**Moderate**
**Qiu et al. 2015 [10]**	**Yes**	**Partial**	**Yes**	**Yes**	**UA**	**Yes**	**Yes**	**Yes**	**Yes**	**No**	**Yes**	**Yes**	**Yes**	**Yes**	**Yes**	**No**	**Moderate**
**Ramos et al 2015 [11]**	**Yes**	**Yes**	**Yes**	**Yes**	**Yes**	**Yes**	**Yes**	**Partial**	**Yes**	**No**	**Yes**	**Yes**	**Yes**	**Yes**	**Yes**	**Yes**	**Moderate**
**Ruan et al 2012 [12]**	**Yes**	**Partial**	**Yes**	**Yes**	**UA**	**Yes**	**No**	**Partial**	**No**	**No**	**Yes**	**Yes**	**Yes**	**Yes**	**Yes**	**Yes**	**Low**
**Shen et al 2013 [13]**	**Yes**	**Partial**	**Yes**	**Yes**	**Yes**	**Yes**	**Partial**	**Yes**	**Yes**	**No**	**Yes**	**Yes**	**Yes**	**Yes**	**Yes**	**Yes**	**High**
**Shen et al 2013 [14]**	**Yes**	**Yes**	**Yes**	**Yes**	**UA**	**Yes**	**Partial**	**Partial**	**Yes**	**No**	**Yes**	**Yes**	**Yes**	**Yes**	**Yes**	**Yes**	**Moderate**
**Shen et al 2014 [15]**	**Yes**	**Partial**	**Yes**	**Yes**	**Yes**	**Yes**	**Partial**	**Yes**	**Yes**	**No**	**Yes**	**Yes**	**Yes**	**Yes**	**Yes**	**Yes**	**High**
**Shen et al 2014 [16]**	**Yes**	**Partial**	**Yes**	**Yes**	**UA**	**Yes**	**Partial**	**Partial**	**Yes**	**No**	**Yes**	**Yes**	**Yes**	**Yes**	**Yes**	**Yes**	**Moderate**
**Shi et al. 2016 [17]**	**Yes**	**Yes**	**Yes**	**Yes**	**UA**	**Yes**	**Partial**	**Partial**	**Yes**	**No**	**Yes**	**No**	**Yes**	**Yes**	**Yes**	**Yes**	**Moderate**
**Wang et al. 2015 [18]**	**Yes**	**Partial**	**Yes**	**Yes**	**UA**	**Yes**	**Partial**	**Partial**	**Yes**	**No**	**Yes**	**Yes**	**Yes**	**Yes**	**Yes**	**Yes**	**Moderate**
**Wu et al 2015 [19]**	**Yes**	**Partial**	**Yes**	**Yes**	**Yes**	**Yes**	**Partial**	**Partial**	**Yes**	**No**	**Yes**	**Yes**	**Yes**	**Yes**	**Yes**	**Yes**	**High**
**Yan et al. 2014 [20]**	**Yes**	**Yes**	**Yes**	**Yes**	**Yes**	**Yes**	**Partial**	**Yes**	**Yes**	**No**	**Yes**	**Yes**	**Yes**	**Yes**	**Yes**	**No**	**Moderate**
**Zhang et al 2013 [21]**	**Yes**	**Yes**	**Yes**	**No**	**Yes**	**Yes**	**Yes**	**Yes**	**Partial**	**No**	**Yes**	**No**	**Yes**	**No**	**Yes**	**Yes**	**Low**

AMSTAR, Assessing the Methodological Quality of Systematic Reviews; SR, Systematic Review; ROB, Risk of Bias; UA, Unable to determine

Supplementary table 6. PCOS specific candidate gene systematic reviews methodology.

Author	What PCOS diagNostic criteria did the SR accept?	Did the SR Note the source of participants for their included studies?	Did the SR have clearly defined control inclusion criteria?	Were controls Not in HWE included in meta-analysis? Was sensitivity analysis performed?
**Metabolic dysfunction**				
Feng et al.[2]	Primary studies where diagNostic criteria was Not defined<br>Rotterdam<br>NIH<br>JSOG	Not described	Not described	Yes HWE was tested.<br>No Sensitivity analysis was performed
Gao et al. [3]	Rotterdam<br>NIH<br>Self-report	Not described	Not described	Yes HWE was tested.<br>Sensitivity analysis was performed
Jia et al.[5]	Rotterdam<br>NIH	Not described	Healthy women without PCOS	Yes HWE was tested.<br>Sensitivity analysis was conducted.
Liao et al.[7]	Rotterdam<br>NIH	Source of controls: Hospital-based and population-based.	Not described	Yes HWE was tested.<br>Sensitivity analysis was conducted.
Liu et al. [8]	Not described	Not described	Not described	Yes HWE was tested.<br>Sensitivity analysis was conducted
Ramos et al.[11]	Not described however all primary studies fulfilled Rotterdam criteria	Not described	Healthy women	No primary studies were excluded if the control group deviated from HWE.
Ruan et al.[12]	Not described	Not described	Unrelated healthy women	Yes HWE was tested.<br>Sensitivity analysis was conducted.
Shen et al.[13]	Rotterdam<br>NIH	Source of controls: Hospital-based and population-based.	Healthy women with Normal menstrual cycles, ovarian morphology, and without history of subfertility treatment	No primary studies were excluded if the control group deviated from HWE.
Shen et al.[14]	Not described	Matched recruitment of cases and controls (Hospital-based and population-based).	Healthy women	No primary studies were excluded if the control group deviated from HWE.
Shen et al.[15]	Rotterdam<br>NIH	Source of controls: Hospital-based and population-based.	Healthy women with Normal menstrual cycles, ovarian morphology, and without history of subfertility treatment	No primary studies were excluded if the control group deviated from HWE.
Shen et al.[16]	Rotterdam<br>NIH	Not described	Not described	No primary studies were excluded if the control group deviated from HWE.
Shi et al.[17]	Not described	Not described	No criteria however stated they included women who were infertile or had other gynaecological conditions in their control group.	Yes HWE was tested.<br>Sensitivity analysis was conducted.
Yan et al.[20]	Not described	Source of controls- Hospital and population based.	Not described	Yes HWE was tested.<br>Sensitivity analysis was conducted.
**Imbalances in Androgens and Gonadotrophins**				
Li et al.[6]	Not described	Not described	Not described	Yes HWE was tested.<br>Sensitivity analysis was conducted.

Pabalan et al. [9]	Not described	Not described	Not described	All control groups were in HWE.
Qiu et al.[10]	Rotterdam<br>Only specific pheNotypes accepted [Hyperandrogenism + irregular cycles + oligo or aNovulation; Hyperandrogenism + Polycystic Ovaries + irregular cycles; Polycystic ovaries + oligo or aNovulation + irregular cycles]	Not described	Age and ethnicity matched healthy women	All control groups were in HWE.
Zhang et al.[21]	Rotterdam<br>NIH<br>Androgen Excess & PCOS Society	Source of controls- Hospital and population based.	Healthy women with proven fertility	Not tested
**Inflammation**				
Chen et al. [1]	Not described	Not described	Healthy women	Yes HWE was tested.<br>Sensitivity analysis was conducted.
Guo et al.[4]	Rotterdam<br>NIH	Not described	Healthy women	Yes HWE was tested.<br>Sensitivity analysis was conducted.
Wang et al.[18]	Not described	Not described	Alive and free from diagNosed PCOS women	Yes HWE was tested.<br>Sensitivity analysis was conducted.
Wu et al.[19]	Rotterdam<br>NIH	Not described	Healthy women	Yes HWE was tested.<br>Sensitivity analysis was conducted.

HWE, Hardy-Weinberg Equilibrium; JSOG, Japanese Society of Obstetrics and Gynaecology; NIH, National Institute of Health; PCOS, Polycystic Ovary Syndrome; SR, Systematic Review.

References

1. Chen, L., et al., *Association between rs1800795 polymorphism in the interleukin-6 gene and the risk of polycystic ovary syndrome: A meta-analysis.* Medicine (Baltimore), 2018. **97**(29): p. e11558.
2. Feng, C., et al., *The association between polymorphism of INSR and polycystic ovary syndrome: a meta-analysis. Int. J. Mol. Sci.*, 2015. **16**(2): p. 2403-25.
3. Gao, J., et al., *The association of DENND1A gene polymorphisms and polycystic ovary syndrome risk: a systematic review and meta-analysis.* Arch Gynecol Obstet, 2016. **294**(5): p. 1073-1080.
4. Guo, R., et al., *Association of TNF-alpha, IL-6 and IL-1beta gene polymorphisms with polycystic ovary syndrome: a meta-analysis. BMC Genet*, 2015. **16**: p. 5.
5. Jia, H., et al., *Associations of adiponectin gene polymorphisms with polycystic ovary syndrome: a meta-analysis. Endocrine*, 2012. **42**(2): p. 299-306.
6. Li, Y., et al., *Polymorphism T->C of gene CYP17 promoter and polycystic ovary syndrome risk: a meta-analysis. Gene*, 2012. **495**(1): p. 16-22.
7. Liao, D., et al., *Association of PON1 gene polymorphisms with polycystic ovarian syndrome risk: a meta-analysis of case-control studies.* J EndocriNol Invest, 2018. **41**(11): p. 1289-1300.
8. Liu, Z., et al., *Effects of ADIPOQ polymorphisms on PCOS risk: a meta-analysis.* Reprod Biol EndocriNol, 2018. **16**(1): p. 120.
9. Pabalan, N., et al., *Associations of Polymorphisms in Anti-Mullerian Hormone (AMH Ile49Ser) and its Type II Receptor (AMHRII -482 A>G) on Reproductive Outcomes and Polycystic Ovary Syndrome: a Systematic Review and Meta-Analysis.* Cell Physiol Biochem, 2016. **39**(6): p. 2249-2261.
10. Qiu, L., J. Liu, and Q.M. Hei, *Association between two polymorphisms of follicle stimulating hormone receptor gene and susceptibility to polycystic ovary syndrome: a meta-analysis. Chin Med Sci J*, 2015. **30**(1): p. 44-50.
11. Ramos, R.B., et al., *Association between rs7903146 and rs12255372 polymorphisms of transcription factor 7-like 2 gene and polycystic ovary syndrome: a systematic review and meta-analysis. Endocrine*, 2015. **49**(3): p. 635-42.
12. Ruan, Y., J. Ma, and X. Xie, *Association of IRS-1 and IRS-2 genes polymorphisms with polycystic ovary syndrome: a meta-analysis.* Endocr J, 2012. **59**(7): p. 601-9.
13. Shen, W., et al., *Calpain-10 genetic polymorphisms and polycystic ovary syndrome risk: a meta-analysis and meta-regression. Gene*, 2013. **531**(2): p. 426-34.

14. Shen, W., et al., *CYP1A1 gene polymorphisms and polycystic ovary syndrome risk: a meta-analysis and meta-regression. Genet Test Mol Biomarkers*, 2013. **17**(10): p. 727-35.
15. Shen, W., et al., *Common polymorphisms in the CYP1A1 and CYP11A1 genes and polycystic ovary syndrome risk: a meta-analysis and meta-regression. Arch Gynecol Obstet*, 2014. **289**(1): p. 107-18.
16. Shen, W.J., et al., *Relationships between TCF7L2 genetic polymorphisms and polycystic ovary syndrome risk: a meta-analysis. Metab Syndr Relat Disord*, 2014. **12**(4): p. 210-9.
17. Shi, X., et al., *Associations of insulin receptor and insulin receptor substrates genetic polymorphisms with polycystic ovary syndrome: A systematic review and meta-analysis. J Obstet Gynaecol Res*, 2016. **42**(7): p. 844-54.
18. Wang, Q., et al., *Meta-analysis of the correlation between IL-6 -174 G/C polymorphism and polycystic ovarian syndrome. J Obstet Gynaecol Res*, 2015. **41**(7): p. 1087-92.
19. Wu, H., K. Yu, and Z. Yang, *Associations between TNF-alpha and interleukin gene polymorphisms with polycystic ovary syndrome risk: a systematic review and meta-analysis. J Assist Reprod Genet*, 2015. **32**(4): p. 625-34.
20. Yan, M.S., et al., *Association of insulin gene variable number of tandem repeats regulatory polymorphism with polycystic ovary syndrome. Hum ImmuNol*, 2014. **75**(10): p. 1047-52.
21. Zhang, T., et al., *Association of the CAG repeat polymorphisms in androgen receptor gene with polycystic ovary syndrome: a systemic review and meta-analysis. Gene*, 2013. **524**(2): p. 161-7.
22. Shea, B.J., et al., *AMSTAR 2: a critical appraisal tool for systematic reviews that include randomised or Non-randomised studies of healthcare interventions, or both.* BMJ, 2017. **358**: p. j4008.

Supplementary Table 7: PRISMA Checklist

Section/topic	#	Checklist item	Reported on page #
**TITLE**			
Title	1	Identify the report as a systematic review, meta-analysis, or both.	1
**ABSTRACT**			
Structured summary	2	Provide a structured summary including, as applicable: background; objectives; data sources; study eligibility criteria, participants, and interventions; study appraisal and synthesis methods; results; limitations; conclusions and implications of key findings; systematic review registration number.	2
**INTRODUCTION**			
Rationale	3	Describe the rationale for the review in the context of what is already known.	3-5
Objectives	4	Provide an explicit statement of questions being addressed with reference to participants, interventions, comparisons, outcomes, and study design (PICOS).	5-6 and supplementary table 1
**METHODS**			
Protocol and registration	5	Indicate if a review protocol exists, if and where it can be accessed (e.g., Web address), and, if available, provide registration information including registration number.	2 and 5

Eligibility criteria	6	Specify study characteristics (e.g., PICOS, length of follow-up) and report characteristics (e.g., years considered, language, publication status) used as criteria for eligibility, giving rationale.	5-7
Information sources	7	Describe all information sources (e.g., databases with dates of coverage, contact with study authors to identify additional studies) in the search and date last searched.	5
Search	8	Present full electronic search strategy for at least one database, including any limits used, such that it could be repeated.	Supplementary table two
Study selection	9	State the process for selecting studies (i.e., screening, eligibility, included in systematic review, and, if applicable, included in the meta-analysis).	5-7
Data collection process	10	Describe method of data extraction from reports (e.g., piloted forms, independently, in duplicate) and any processes for obtaining and confirming data from investigators.	5-7
Data items	11	List and define all variables for which data were sought (e.g., PICOS, funding sources) and any assumptions and simplifications made.	5-7
Risk of bias in individual studies	12	Describe methods used for assessing risk of bias of individual studies (including specification of whether this was done at the study or outcome level), and how this information is to be used in any data synthesis.	6
Summary measures	13	State the principal summary measures (e.g., risk ratio, difference in means).	6
Synthesis of results	14	Describe the methods of handling data and combining results of studies, if done, including measures of consistency (e.g., I^2) for each meta-analysis.	N/A

Section/topic	#	Checklist item	Reported on page #
Risk of bias across studies	15	Specify any assessment of risk of bias that may affect the cumulative evidence (e.g., publication bias, selective reporting within studies).	Supp table 4
Additional analyses	16	Describe methods of additional analyses (e.g., sensitivity or subgroup analyses, meta-regression), if done, indicating which were pre-specified.	N/A
**RESULTS**			
Study selection	17	Give numbers of studies screened, assessed for eligibility, and included in the review, with reasons for exclusions at each stage, ideally with a flow diagram.	Figure 1
Study characteristics	18	For each study, present characteristics for which data were extracted (e.g., study size, PICOS, follow-up period) and provide the citations.	Sup table 3 and 5
Risk of bias within studies	19	Present data on risk of bias of each study and, if available, any outcome level assessment (see item 12).	Supp table 4
Results of individual studies	20	For all outcomes considered (benefits or harms), present, for each study: (a) simple summary data for each intervention group (b) effect estimates and confidence intervals, ideally with a forest plot.	Figure 3,4,5,

			tables 1 & 2, Sup table 3
Synthesis of results	21	Present results of each meta-analysis done, including confidence intervals and measures of consistency.	N/A
Risk of bias across studies	22	Present results of any assessment of risk of bias across studies (see Item 15).	N/A
Additional analysis	23	Give results of additional analyses, if done (e.g., sensitivity or subgroup analyses, meta-regression [see Item 16]).	N/A
**DISCUSSION**			
Summary of evidence	24	Summarize the main findings including the strength of evidence for each main outcome; consider their relevance to key groups (e.g., healthcare providers, users, and policy makers).	Pg 9-14. Figure 3,4,5, tables 1 & 2, Sup table 3.
Limitations	25	Discuss limitations at study and outcome level (e.g., risk of bias), and at review-level (e.g., incomplete retrieval of identified research, reporting bias).	15 and sub table 4
Conclusions	26	Provide a general interpretation of the results in the context of other evidence, and implications for future research.	16-21
**FUNDING**			
Funding	27	Describe sources of funding for the systematic review and other support (e.g., supply of data); role of funders for the systematic review.	2

From: Moher D, Liberati A, Tetzlaff J, Altman DG, The PRISMA Group (2009). Preferred Reporting Items for Systematic Reviews and Meta-Analyses: The PRISMA Statement. PLoS Med 6(6): e1000097. doi:10.1371/journal.pmed1000097

For more information, visit: **www.prisma-statement.org**.
